# Socioeconomic position over the life course from childhood and smoking status in mid-adulthood: results from a 25-year follow-up study

**DOI:** 10.1186/s12889-019-6483-0

**Published:** 2019-02-08

**Authors:** Jing Tian, Seana Gall, Kira Patterson, Petr Otahal, Leigh Blizzard, George Patton, Terry Dwyer, Alison Venn

**Affiliations:** 1 Menzies Institute for Medical Research, University of Tasmania, 17 Liverpool Street, Hobart, Tasmania 7000 Australia; 2 Murdoch Childrens Research Institute, Melbourne, Victoria Australia; 3 The George Institute for Global Health, University of Oxford, Wellington Square, Oxford, UK

**Keywords:** Socioeconomic position, Social mobility, Smoking, Child, Adult, Prospective studies

## Abstract

**Background:**

It remains unclear how life course socioeconomic position (SEP) variations impact later smoking status. We aimed to investigate the associations using a novel methodology – a structured regression framework and to explore the potential underlying mechanisms.

**Methods:**

Data were from an Australian national cohort (*n* = 1489). SEP was measured in childhood (aged 7–15 years), young- (aged 26–36 years) and mid-adulthood (aged 31–41 years), including highest parental occupation in childhood and self-occupation in young- and mid-adulthood. Smoking status was self-reported in mid-adulthood. Four smoking-related variables in childhood including exposure to parental smoking, smoking experimentation, self-rated importance to be a non-smoker and intention to smoke were tested as potential mediators. A structured life course modelling approach was used to select the best-fit life course model(s). The log multinomial model was used to estimate the smoking risk in mid-adulthood with never smokers as the excluded category.

**Results:**

63.6% of participants were classified as stable non-manual occupation across the life course from childhood. The sensitive period and the accumulation model described the data equally as well as the saturated model. In the sensitive period model, compared to the non-manual group, those who had highest parental occupation of manual had a 21% lower risk of being former smokers and a 32% greater risk of being current smokers in mid-adulthood, and those who were occupied manually in mid-adulthood reported a 55% greater risk of being current smokers in mid-adulthood. In the accumulation model, compared to those who consistently reported non-manual occupations across the life course, those with manual occupations for longer had higher risk of being current smokers in mid-adulthood, with a 43% risk increase per time point in a manual occupation. Exposure to parental smoking and intention to smoke during childhood explained up to 40.2% of the excess risk of being current smokers in mid-adulthood associated with manual occupations in the sensitive period and the accumulation model.

**Conclusions:**

Childhood, young- and mid-adulthood are all important, but SEP in childhood and mid-adulthood may be of more importance in determining mid-adulthood smoking status. Exposure to parental smoking and intention to smoke in childhood seems to moderately mediate the associations.

**Electronic supplementary material:**

The online version of this article (10.1186/s12889-019-6483-0) contains supplementary material, which is available to authorized users.

## Background

Tobacco smoking disproportionately affects groups of low socioeconomic position (SEP) [1]. In the past few decades, there has been an overall downward trend in smoking prevalence across most demographic groups in countries with advanced tobacco control programs [2, 3]. However, the declines are generally greater in less disadvantaged groups, contributing to a widening disparity in smoking by SEP [2, 4]. For example, in Australia, the gap in smoking prevalence between low and high SEP groups among people aged 14 years and over widened from 8.6% in 1998 [5] to 13.2% in 2013 [6].

Of importance to the role of SEP in smoking behaviour is the reasons for the association. Children from socioeconomically disadvantaged families are more often exposed to parental smoking [7], have more favourable attitudes toward smoking [8], a greater intention to smoke [9] and early smoking experimentation [10] than those from less disadvantaged backgrounds. In turn, these factors are associated with increased risk of future smoking [7, 11–16]. Few longitudinal studies have explored the extent to which these factors might account for the SEP differences in later smoking [7] and none have taken into account the impact of adult SEP, which is closely related to childhood SEP and adulthood smoking [1].

Understanding whether SEP at different life stages differentially impacts later smoking and the underlying mechanisms may help to inform policies to reduce the high prevalence in low SEP groups. Various models have been proposed to describe how exposures such as SEP may operate over the life course [17]. The critical period model assumes the effect of SEP is important at a limited time window and that there is no influence outside this time period. The sensitive period model assumes the effect of SEP is stronger at one time period than at other times. The accumulation of risk model assumes SEP affects the outcome cumulatively and equally over the life course. Social mobility models vary across different definitions. The intra-generational (adult) mobility model assumes that any downwards change in SEP in adulthood would be harmful to the outcome and any upwards mobility in adulthood would be beneficial, independent of childhood social background. Any mobility model hypothesises that all downward changes in the life course are equally harmful to the outcome and all upward shifts are equally beneficial. There is evidence that these models are useful for understanding the development of health across the life course. Using data on adult body mass index (BMI) and SEP measured once in childhood and twice in adulthood from the Medical Research Council National Survey of Health and Development study, Mishra et al. [17] concluded that only considering one life course model may produce misleading results and recommended considering all possible models in such analyses. A systematic review of models of life course socioeconomic factors also recommended to test multiple life course models and use multiple SEP measures in one sample in future analyses [18].

Utilising longitudinal data at different life stages, several studies have tried to understand the relationship of SEP across the life course and smoking status in later life. These were limited in that they included only one potential life course model such as the critical period model (only childhood reflected by parental SEP) [19, 20], the sensitive period model [21–23], the social mobility model [24], and the accumulation model [25, 26]. However, no study has investigated how all of the possible life course models might describe the association between SEP and smoking in later life in one sample. The aim of this study was to examine the importance of timing and duration of exposure to low SEP, and mobility in SEP, for mid-adulthood smoking in an Australian national cohort. We also investigated whether smoking-related variables in childhood mediated the relationship.

## Methods

### Participants

The Childhood Determinants of Adult Health (CDAH) study includes 20- and 25-year follow-ups of Australian school children and adolescents aged 7–15 years who participated in the 1985 Australian Schools Health and Fitness Survey (ASHFS) (*n* = 8498, baseline, also referred to as “childhood”) [27]. At baseline, a two-stage probability sampling framework was used to achieve a nationally representative sample. The first stage was the selection of schools (government, Catholic, and independent) with a probability proportional to size (*n* = 109, 90.1% response rate), and the second stage was the random sampling of 10 boys and girls from each age strata within schools (*n* = 8498, 67.5% response rate).

During 2002–04, we traced 6840 subjects and 5170 of them agreed to participate in the follow-up study and completed a brief postal or telephone questionnaire. About two years later (2004–06), 3521 individuals aged 26–36 years completed the first follow-up (CDAH-1, herein also referred to “young adulthood”) and 2410 of them also attended one of 34 clinics held in each state and territory of Australia for physical measurements. The second follow-up (CDAH-2, herein also referred to “mid-adulthood”) was conducted in 2009–11, when participants were aged 31–41 years. A total of 2815 participants completed a postal questionnaire or a computer–assisted telephone interview.

At baseline, the Directors of Education in each state granted approval, and consent was obtained from children and parents. At CDAH-1 and 2, the Southern Tasmanian Health and Medical Ethics Committee approved the study protocol and written informed consent was obtained from participants.

### SEP assessment over the life course

The Australian Standard Classification of Occupations was used to assess the occupation level at three time points [28]. It classifies occupation into nine levels from manager or administrator to labourer or related worker which were regrouped into non-manual (managers, professionals and white collar) and manual (blue collar) SEP groups, similar to measures used in several other epidemiological studies [29, 30]. A few percent of participants or their parents were not in the labour force. Their SEP was determined by the highest own or parental education, with post-school qualification (any university degree or trade/vocational training) as non-manual group and without (year 12 or less) as manual group.

Baseline parental occupation was retrospectively reported by participants at CDAH-1. For each parent, participants reported the main occupation of their father/mother (or other male/female who lived with them and was like a father/mother to them) for most of the time when they were growing up until 12 years old. The level of occupation for whichever parent had the highest was used as the indicator of childhood SEP. We interpreted this as an indicator of the early life home environment of the child. Participants self-reported their occupation at CDAH-1 and 2.

### Smoking status assessment

Participants were classified into never, former and current smokers according to their responses to two questions at CDAH-2 [31]. The first question asked “Over your lifetime, have you smoked at least 100 cigarettes, or a similar amount of tobacco?” Participants answering “yes” were classified as ever smokers, and those answering “no” as never smokers. Ever smokers were then asked the second question “How often do you now smoke cigarettes, cigars, pipes or any other tobacco products?” Participants who answered “not at all” were classified as former smokers and those who answered “daily” or “at least once a week” or “less than weekly” were classified as current smokers.

### Potential mediators in childhood

Children and adolescents aged 9–15 years completed questionnaires in small groups with a study data collector. Children under 9 years of age were deemed too young to complete the questionnaires reliably. Children and adolescents reported whether their mother or father smoked at home. Exposure to parental smoking was coded as “Neither parents smoke”, “One parent smokes” and “Both parents smoke” [32]. Smoking experimentation was collected using a question “Have you ever smoked even part of a cigarette?”. Children and adolescents could respond “no”, “yes, a few puffs”, “yes, I have smoked fewer than 10 cigarettes in my life” and “yes, I have smoked more than 10 cigarettes in my life” [12]. The latter three categories were collapsed into one group as with childhood smoking experimentation. Importance to be a non-smoker was assessed by asking “In your opinion how important is being a non-smoker to you”. They could answer “very important”, “of some importance”, “of little importance” or “not important”. Information on whether they would be smoking this time next year was also collected. Possible answers included “Yes”, “No” and “Don’t know”. The English language version of questions used to collect the data of importance to be a non-smoker and intention to smoke are shown in Additional file 1.

### Statistical analysis

The log multinomial model, which estimated relative risks (RRs) and 95% confidence intervals (CIs) with multiple attributes [33], was used to estimate the risk of smoking in mid-adulthood with never smokers as the excluded category. We did not separate men and women for analyses as tests of interaction revealed no evidence of significant difference.

A structured modelling approach was used to test which life course model(s) best fit the data [17]. This framework compares a set of nested models to a saturated model. The saturated model included SEP at three time points and all two and three-way interactions. The sensitive period model simultaneously included SEP at three time points. The critical period model consisted of three separate models for each SEP time point. The accumulation model was tested by summing the number of times that a person experienced low SEP across the early life span to form an overall score ranging from 0 to 3, which was then used as the exposure in log multinomial models. Social mobility was categorised into three groups (stable manual or non-manual (or variable in any mobility model), moving upwards, and moving downwards), which was determined by the SEP at CDAH-1 and 2 in the intra-generational (adult) mobility model and by the SEP at three time points for any mobility model. Model specifications and constrains are described in detail in Additional file 2**:** Table S1.

Likelihood ratio tests were used to examine whether the fit of each nested model was as good as the fully saturated model. A large *P*-value (> 0.10) indicates no evidence of statistically significant difference between the tested nested model and the fully saturated model. Two or more nested life course models might fit the data similarly to the fully saturated model [34]. This structured regression framework has been widely used in the literature [35–37].

Potential mediators in childhood were chosen according to a priori causal knowledge and univariable analyses. Only variables which significantly associated with smoking status at CDAH-2 and SEP at baseline were added into the best-fitting life course model(s). The percent excess risk explained by the tested potential mediator was obtained by a ratio where the numerator included the difference in RRs between models before (RRu) and after adding the potential mediator (RRa), and the denominator included the unadjusted excess risk (% excess risk explained = (RRu – RRa)/(RRu – 1) * 100) [38]. Approximately 20% of participants missed information on one or more potential mediators so multiple imputation (MI) by chained equations was used [39], with the number of imputations being 20 [40].

The first sensitivity analysis was conducted by defining SEP according to an area-level measure – socio-economic indexes for areas (SEIFA), a product developed by the Australian Bureau of Statistics that ranks residential area in Australia based on socioeconomic advantage and disadvantage and consists of four indexes [41]. The Index of Relative Socioeconomic Disadvantage (IRSD) was the most commonly used in the literature and was used in this study. IRSD focuses on relative disadvantage and is derived from variables such as income, educational attainment, housing tenure and car availability. Participants were assigned to a score based on census collection area of their residence place. A low score indicates a high proportion of relatively disadvantaged people in an area and a high score indicates a relative lack of disadvantage in general. In order to limit the number of alternative pathways over the life course, IRSD was dichotomised into relatively disadvantaged and a lack of relative disadvantage group based on the median value. The second sensitivity analysis was performed using combined MI and inverse probability weight (IPW) [42] to address loss to follow-up.

Statistical analyses were performed with STATA 15.0 (Stata Corp, College Station, Texas).

## Results

### Participant characteristics

**Fig. 1:**
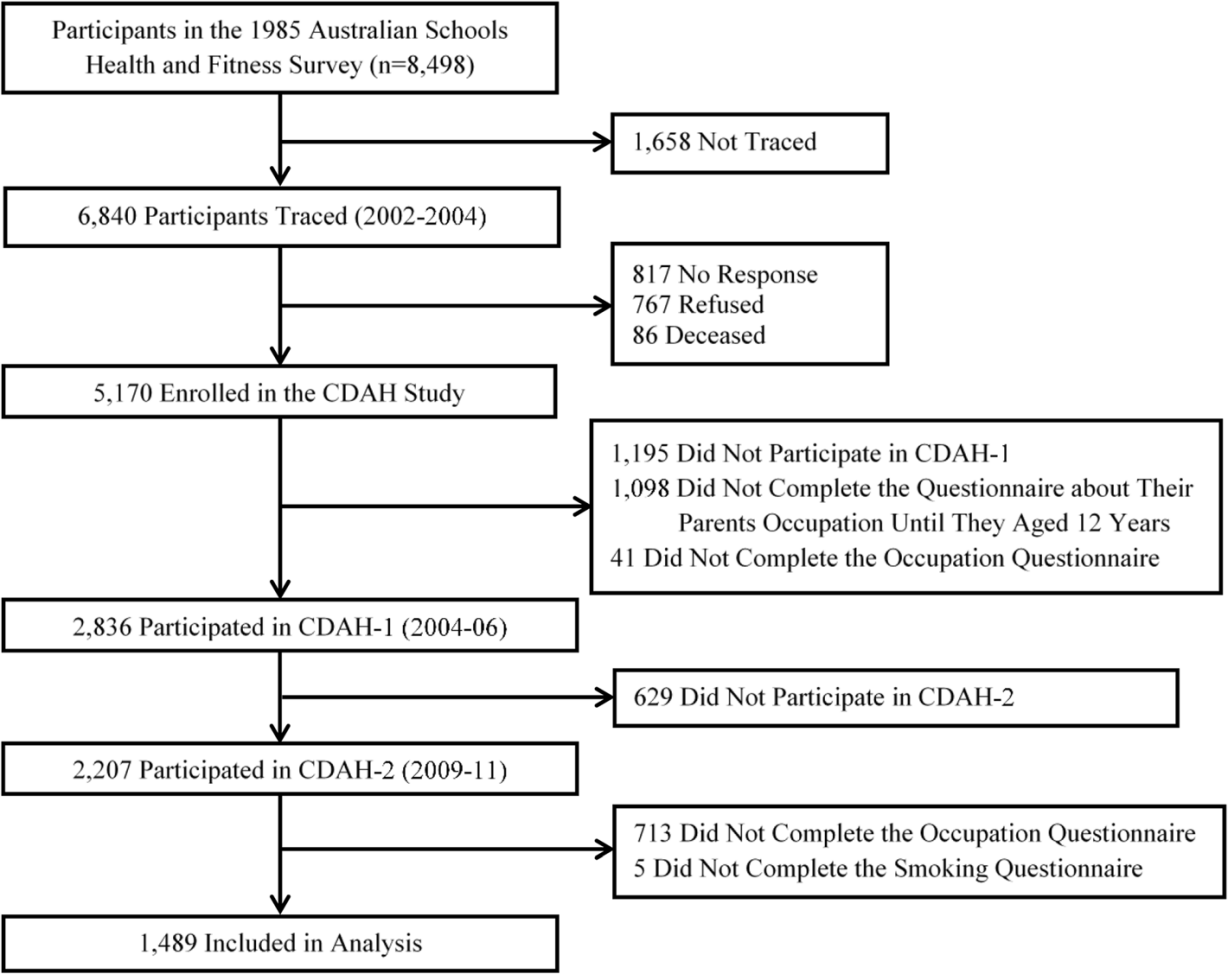
Flow chart of recruitment and retention of participants for Childhood Determinants of Adult Health Study, Australia, 1985–2011

Flow chart of recruitment and retention of participants is presented in Fig. 1. A total of 1489 participants were included in the final analyses. Their sociodemographic characteristics at CDAH-2 and SEP trajectories across the early life span are shown in Table 1. 63.6% of participants were classified as stable non-manual occupation across the early life span.

**Table 1:** Characteristics of participants at the final follow-up (CDAH-2, 2009–11) and socioeconomic trajectories over the early life course, Childhood Determinants of Adult Health study, Australia^*^

Sociodemographic characteristics			Total (*n* = 1489)
Age (years), Mean (SD)			36.5 (2.6)
Males, % (n)			36.7 (546)
Marital status, % (n)			
Single			14.4 (214)
Married/living as married			81.4 (1211)
Separated/divorced/widowed			4.2 (62)
Weight status^†^, % (n)			
Normal (< 25)			50.3 (708)
Overweight (25–29.9)			32.9 (463)
Obese (≥30)			16.8 (237)
Smoking status, % (n)			
Never smokers			60.7 (904)
Former smokers			25.2 (375)
Current smokers			14.1 (210)
Education, % (n)			
With post-school qualification			80.2 (1187)
Without post-school qualification			19.9 (294)
SEIFA disadvantage, % (n)			
A relative lack of disadvantage			51.9 (768)
Relatively disadvantaged			48.1 (711)
SEP characteristics			
Baseline, % (n)			
Non-manual (0)			76.7 (1142)
Manual [1]			23.3 (347)
CDAH-1, % (n)			
Non-manual (0)			82.8 (1233)
Manual [1]			17.2 (256)
CDAH-2, % (n)			
Non-manual (0)			84.8 (1263)
Manual [1]			15.2 (226)
SEP trajectories across three time periods, % (n)			
Baseline	CDAH-1	CDAH-2	
0	0	0	63.6 (947)
1	0	0	15.1 (225)
0	1	0	3.8 (56)
0	0	1	2.8 (42)
1	1	0	2.4 (35)
1	0	1	1.3 (19)
0	1	1	6.5 (97)
1	1	1	4.6 (68)
Accumulation model: number of times manual, % (n)			
0			63.6 (947)
1			21.7 (323)
2			10.1 (151)
3			4.6 (68)
Social mobility model^‡^			
Intra-generational (adult) mobility, % (n)			
Stable (non-)manual			89.8 (1337)
Moving downwards			4.1 (61)
Moving upwards			6.1 (91)
Any mobility, % (n)			
Stable (non-)manual/variable			73.2 (1090)
Moving downwards			9.3 (139)
Moving upwards			17.5 (260)

CDAH: childhood determinants of adult health; SEIFA: socioeconomic index for areas; SEP: socioeconomic position

^*^ Sample size varied because of missing data (range, 1405-1489). Some summed proportions not 100% due to rounding off; non-manual occupation level denoted by 0 and manual occupation level denoted by 1

^†^ Defined by body mass index

^‡^ The intra-generational (adult) mobility model assumes that any downwards change in SEP in adulthood would be harmful to the outcome and any upwards mobility in adulthood would be beneficial, independent of childhood social background. Any mobility model hypothesises that all downward trend changes in the life course are equally harmful to the outcome and all upward shifts are equally beneficial

Using baseline (1985 ASHFS) characteristics, compared with those lost to follow-up, those who participated in the follow-up study were more often female (63% vs. 47%). No statistically significant differences were observed in age (both 11.1 years), BMI (18.1 vs. 18.2 kg/m^2^), Australian-born (93.5% vs. 94.0%), having a very good self-rated health status (37.1% vs. 36.0%), highest parental occupation non-manual (76.6% vs. 73.6%), highest parental education with post-school qualification (62.1% vs. 60.3%), and area-level disadvantage (1st quartile (highest SEP), 26.1% vs. 26.4%).

Using CDAH-2 (2009–11) characteristics, compared with the Australian general population of adults aged 35–44 years old, a higher percentage of CDAH participants were married or living as married (81.4% vs. 74.1%) and were employed as professionals and/or managers (57.6% vs. 39.2%) [43], and a lower percentage were classified as overweight or obese (BMI ≥ 25 kg/m^2^) (49.8% vs. 64.9%) [44].

### Life course SEP and adult smoking status

Table 2 presents the percentage and number of participants by smoking status in CDAH-2 and SEP life course models. Occupation at each of the three time points and its trajectories were significantly associated with CDAH-2 smoking status.

**Table 2:** Smoking status at the final follow-up (CDAH-2, 2009–11) by SEP life-course models, Childhood Determinants of Adult Health study, Australia^*^

Life course model	Never smokers (*n* = 904)	Former smokers (*n* = 375)	Current smokers (*n* = 210)	*P*-value
Individual time period (sensitive/ critical period model)				**0.023**
Baseline, % (n)				
Non-manual	77.3 (699)	79.2 (297)	69.5 (146)	
Manual	22.7 (205)	20.8 (78)	30.5 (64)	
CDAH-1, % (n)				**< 0.001**
Non-manual	86.5 (782)	80.8 (303)	70.5 (148)	
Manual	13.5 (122)	19.2 (72)	29.5 (62)	
CDAH-2, % (n)				**< 0.001**
Non-manual	88.1 (796)	83.7 (314)	72.9 (153)	
Manual	12.0 (108)	16.3 (61)	27.1 (57)	
Accumulation model: No. of times manual, % (n)				**< 0.001**
0 time manual	66.7 (603)	64.3 (241)	49.1 (103)	
1 time manual	21.9 (198)	19.7 (74)	24.3 (51)	
2 times manual	8.0 (72)	11.5 (43)	17.1 (36)	
3 times manual	3.4 (31)	4.5 (17)	9.5 (20)	
Social mobility model^†^, % (n)				
Intra-generational (adult) mobility				**0.002**
Stable (non-)manual	91.8 (830)	89.1 (334)	82.4 (173)	
Moving downwards	3.3 (30)	4.0 (15)	7.6 (16)	
Moving up wards	4.9 (44)	6.9 (26)	10.0 (21)	
Any mobility				**0.042**
Stable (non-)manual/variable	73.8 (667)	74.1 (278)	69.1 (145)	
Moving downwards	7.7 (70)	10.7 (40)	13.8 (29)	
Moving up wards	18.5 (167)	15.2 (57)	17.1 (36)	

CDAH: childhood determinants of adult health

^*^ Some summed proportions not 100% due to rounding off

All bolded *P*-values are statistically significant at the 0.05 level

^†^ The intra-generational (adult) mobility model assumes that any downwards change in SEP in adulthood would be harmful to the outcome and any upwards mobility in adulthood would be beneficial, independent of childhood social background. Any mobility model hypothesises that all downward trend changes in the life course are equally harmful to the outcome and all upward shifts are equally beneficial

The results by life course model fit are described in Table 3. The sensitive period model and the accumulation model described the data equally as well as the saturated model which is reflected by the high *P*-values. The critical period and social mobility models showed particularly poor fit as the P-value was less than 0.10. Therefore, the sensitive period and the accumulation models were selected for further analyses. As shown in Model 1 **(**Table 4**)**, in the sensitive period model, compared to the non-manual group, those who had highest parental occupation of manual in childhood had a 21% lower risk of being former smokers at CDAH-2 when they were 31–41 years and a 32% greater risk of being current smokers at CDAH-2, and those who were occupied manually at CDAH-2 reported a 55% greater risk of being current smokers at CDAH-2. In the accumulation model, compared to those who consistently reported non-manual occupation across the life course, those who were exposed to manual occupations for longer had higher risk of being current smokers at CDAH-2, with a 43% risk increase per time point in manual occupation. These results suggest that all time points are important, but SEP in childhood and CDAH-2 may be more important to determine smoking status in CDAH-2.

**Table 3:** *P*-values from likelihood ratio tests for associations between SEP variations across the life course from childhood determined by occupation (or parental occupation) and smoking status in mid-adulthood, comparing each life course model with the saturated model, Childhood Determinants of Adult Health study, Australia^*^

Life course model	Model fit (compared to the saturated model)
	*P*-value
No effect model	< 0.001
Sensitive period model^†^	**0.332**
Critical period model	
Manual, baseline	< 0.001
Manual, CDAH-1	0.051
Manual, CDAH-2	0.028
Accumulation model, No. of times manual	**0.117**
Social mobility model	
Intra-generational mobility	< 0.001
Any mobility	0.007

CDAH: childhood determinants of adult health

^*^ All models were adjusted for age and sex at CDAH-2

^†^ Life course models in bold are the best-fitting models

**Table 4:** Effects of exposure to parental smoking and intention to smoke in childhood on the relationship of SEP across the early life course and mid-adulthood (CDAH-2, 2009–11) smoking in the best-fitting life course models, Childhood Determinants of Adult Health study, Australia^*^

Best-fitting life course models	Model 1 (adjusted for age + sex)	Model 2 (Model 1 + exposure to parental smoking)		Model 3 (Model 1 + intention to smoke in the following year)	
	RR (95% CI)	RR (95% CI)	Excess risk explained^†^, %	RR (95% CI)	Excess risk explained^†^, %
Sensitive period model					
Former smokers at CDAH-2^§^					
Manual, baseline	**0.79 (0.63, 0.98)**	**0.78 (0.63, 0.98)**	−2.6	**0.76 (0.60, 0.95)**	−14.6
Manual, CDAH-1	1.25 (0.93, 1.68)	1.24 (0.93, 1.67)	3.0	1.20 (0.89, 1.60)	22.1
Manual, CDAH-2	1.00 (0.73, 1.37)	0.99 (0.72, 1.35)	> 100^‡^	0.97 (0.71, 1.33)	> 100^‡^
Current smokers at CDAH-2^§^					
Manual, baseline	**1.32 (1.00, 1.73)**	1.21 (0.93, 1.58)	33.0	1.19 (0.89, 1.59)	40.2
Manual, CDAH-1	1.42 (0.95, 2.11)	1.43 (0.98, 2.10)	−3.4	1.24 (0.83, 1.86)	42.3
Manual, CDAH-2	**1.55 (1.04, 2.30)**	1.42 (0.97, 2.09)	23.0	**1.50 (1.01, 2.25)**	7.8
Accumulation model, No. of times manual					
Former smokers at CDAH-2^§^	1.01 (0.91, 1.12)	1.00 (0.90, 1.11)	> 100^‡^	0.97 (0.87, 1.07)	> 100^‡^
Current smokers at CDAH-2^§^	**1.43 (1.27, 1.61)**	**1.36 (1.21, 1.53)**	16.0	**1.32 (1.17, 1.48)**	20.6

RR: relative risk; CI: confidence interval; CDAH: childhood determinants of adult health

^*^ About 20% participants missed childhood potential mediators’ data. Multiple imputation by chained equations was used to deal with the missing data, with 20 imputations

^†^ The percent excess risk explained = (RRu – RRa)/(RRu – 1) * 100. RRu was the average RR in Model 1. RRa was the average RR in Model 2 or 3

^‡^ Estimation of excess risk as a percentage of Model 1 RR is unreasonable when the RR in Model 1 was extremely close to 1

^§^ Non-smokers were the excluded category for the outcome

Bold RRs (95% CIs) indicate statistically significant results in the best-fitting life course models

### Examination of potential mediators in best-fit life course models

Exposure to parental smoking and intention to smoke in the following year in childhood were significantly associated with mid-adulthood (CDAH-2) smoking status and childhood SEP. Among the 28 children who intended to smoke in the following year, 57.1% [16] had at least one parent who smoked. These two variables were therefore tested in the mediation analysis (Table 4). In the sensitive period model, further adjustment for exposure to parental smoking in childhood accounted for 33.0% excess risk of being current smokers at CDAH-2 among people whose highest parental occupation was manual in childhood and 23.0% excess risk among people who occupied manually at CDAH-2 (Model 2). Further adjustment for intention to smoke in the following year in childhood moderately reduced the risk of being current smokers at CDAH-2 among people whose highest parental occupation was manual in childhood (40.2%) (Model 3). In the accumulation model, further adjustment for exposure to parental smoking and intention to smoke reduced the higher risk of being current smokers at CDAH-2 among people who had been exposed to manual occupation, by 16.0 and 20.6% per time individually.

### Sensitivity analyses

When defining SEP according to area-level disadvantage, the sensitive period model provided the best fit compared to the saturated model (Additional file 2**:** Table S2), in which living in a relatively disadvantaged area in childhood significantly decreased the probability of being former smokers at CDAH-2 and living in a relatively disadvantaged area at CDAH-1 and -2 significantly increased the risk of being current smokers at CDAH-2. The associations between SEP variations across the life course and smoking status in mid-adulthood after applying combined MI & IPW were very similar to the original results (Additional file 2: Table S3).

## Discussion

This is the first study to examine the relationship between SEP trajectories over the life course and smoking later life using a structured regression framework to examine a series of theoretical life course models. For individual-level SEP, the sensitive period model and the accumulation model best fit the data. The risk of being a current smoker in mid-adulthood was higher in those exposed to low SEP in childhood and mid-adulthood and for those exposed with greater cumulative exposure. For area-level SEP, the model that best described the data was the sensitive period model in which the smoking risk was highest in those exposed to low SEP in early and mid-adulthood. This association was moderately explained by exposure to parental smoking and intention to smoke in childhood.

The sensitive period model was supported by our individual- and area-level SEP data. Being exposed to low individual-level SEP in the “sensitive periods” of childhood and mid-adulthood and to low area-level SEP in young- and mid-adulthood increased the risk of being a current smoker in mid-adulthood when SEP at all three life stages were mutually adjusted. There is considerable evidence that smoking in adulthood is influenced by childhood and adulthood socioeconomic disadvantage [7, 18, 45, 46]. For example, according to Kestila et al. [45], young adults whose parents had the lowest educational attainment were about five times more likely to be a daily smoker than those with parents in the highest education category.

The results for individual-level SEP shows strong support for the accumulation model for adult smoking. This finding is consistent with the study by Smith et al. [25] which assessed the influence of SEP over three life stages on risk factors of cardiovascular disease including smoking among 5766 men. They revealed a positive graded association between the number of time periods belonging to manual occupation social class and the risk of being current smokers. There is also evidence for a similar association in women, where belonging to a manual occupation social class in both childhood and adulthood increased the odds of being current smokers by 75% compared with staying in non-manual social class at both time points [47]. Nevertheless, it should be noted that direct comparison with other studies is limited because their analyses were not framed in terms of life course models.

We found inconsistent results when using socioeconomic indicators at the individual and area levels: both the sensitive period model and the accumulation model were found to best fit the data when using individual-level SEP where only the sensitive period model fit when using area-level SEP. Previous evidence of the validity of using area-based SEP measures as proxies of individual-level indicators is conflicting [48, 49]. One of the possible explanations is the different constructs of area and individual-level socioeconomic measures [48]. Using data from three large population-based epidemiologic studies, Diez and colleagues reported that area and individual-level indicators were somewhat correlated but actually provided complementary information on living circumstances [48]. Presence of contextual area effects may help explain discrepancies between area- and individual-based estimates of socioeconomic differences in smoking [48]. This involves mechanisms through which contextual effects of area on smoking could be mediated, including greater risk of being exposed to smoking and greater availability of places that sell cigarettes in low SEP areas [1]. Another possible reason is that the SEP of area as a whole will not always represent the SEP of individuals (the “ecological fallacy”). Some individuals with a higher individual-level SEP may reside in a relatively disadvantaged area and in contrast, some individuals with a lower individual-level SEP may live in an area which relatively lacks disadvantage [50].

As expected, the observed higher risk of smoking in mid-adulthood among people exposed to low SEP in childhood and mid-adulthood and for a greater number of periods was partially explained via exposure to parental smoking during childhood. This finding is in line with past studies by Paul et al. [12] and Fergusson et al. [7], which concluded that smoking in adulthood was predicted by exposure to parental smoking that could account for over 25% of the relationship between childhood social background and later smoking [7]. In the current study intention to smoke, self-rated importance to be a non-smoker and smoking experimentation in childhood were explored as potential explanations the SEP gradient in smoking for the first time. Our results suggest childhood socioeconomic disadvantage influenced smoking in mid-adulthood partially through intention to smoke in childhood.

Our findings reiterate the important roles of exposure to parental smoking and intention to smoke in childhood in the relationship of SEP across the early life span and smoking in mid-adulthood. The increased risk of smoking in offspring of people that smoke along with the well-established health problems and illnesses of second-hand smoke may be used to encourage parents and those who will become parents to quit smoking. This approach is likely to have a large impact as parents are strongly motivated to adopt healthy behaviours for the sake of their children [51]. For adult smokers in low SEP, increasing tobacco taxes are believed to have the greatest potential to achieve reduction in smoking [52, 53].

Some limitations should be considered. First, self-report could result in the misclassification of smoking status; however, it is most likely that people have under-reported smoking which would likely mean we have underestimated the effect of SEP on current smoking [54]. Second, dichotomising SEP is very simplified but necessary for the modelling framework. We could not explore whether there was a gradient of effects across socioeconomic levels. Third, we did not know the full duration of exposure so the accumulation model in this study does not refer to the exact length of exposure to low or high SEP. Fourth, we were somewhat limited in the approaches we could use in mediation analyses because our potential explanatory variables were measured at a single point in time that was concurrent with one of our exposures – childhood SEP. This precludes using a method such as path analysis with structural equation modelling where it is recommended to use temporally separate exposures, mediators and outcomes. Fifth, our mediation results should be interpreted with caution since the traditional approach to mediation analyses we have used relies on fairly strong assumptions including control for mediator-outcome, exposure-mediator, and exposure-induced mediator-outcome confounding to be interpreted causally [55]. Failure to control for these assumptions may produce flawed results [38, 55]. In our study, genetics and particular personality traits (i.e. extraversion, neuroticism and conscientiousness) that affect both early smoking experimentation and established smoking patterns in adulthood, are potential mediator-outcome confounders [56, 57]. Unfortunately, genetic data were not available and adjusting for personality traits measured by the NEO-Five Factor Inventory in CDAH-1 showed no evidence of mediator-outcome confounding. No exposure-mediator interaction was observed for exposure to parental smoking and a moderate interaction was present between childhood SEP and children’s intention to smoke. Therefore, the natural direct effect which incorporated the interaction effects was estimated using Richiardi and colleagues’ approach [38]. Due to the low prevalence of intention to smoke (2.3%) in our cohort, the estimated natural direct effect is almost identical to the direct effect shown in Table 4, indicating our conclusions are largely unchanged by this interaction. The smoking behaviour of peers has been identified as a strong predictor of both intention to smoke and smoking uptake among adolescents [57, 58] but is unlikely to act as a substantial exposure-induced mediator-outcome confounder. This is because of its weak association with childhood SEP [58]. The direct effect estimated in Table 4 (Model 3 with adjustment for intention to smoke) might be overestimated, depending on the strength of the independent relationship between peers smoking and mid-adulthood smoking.

The strengths of this study include its large national sample, the 25 years follow-up period, the use of a novel methodology – a structured regression framework to modelling the effects of binary exposure variables over the life course and the efforts to explore the underlying mechanisms. Although several studies have examined the association of SEP and smoking status using a life course approach, none of them has tested multiple life course models in the same sample. As concluded by Pollitt and colleagues in a systematic review [18], analyses using data followed from childhood to adulthood, multiple SEP measures and multiple life course designs within the same sample offer the best approach to test which theories best describe the association between life course SEP and the outcome. The structured regression framework we used to compare a set of nested models to an all-inclusive (fully saturated) model is an improvement over traditional regression models in which results are interpreted from a single pre-specified hypothesis without considering the merits of alternative life course hypotheses. For example, if we only considered a single model, such as the accumulation model, we might conclude that there is evidence for its fit. However, the sensitive period model would not be identified even though it fits the data just as well as the fully saturated model with both individual- and area-level SEP.

To conclude, childhood, young- and mid-adulthood are all important, but SEP in childhood and mid-adulthood may be of more importance in determining smoking status in mid-adulthood. Exposure to parental smoking and intention to smoke in childhood seems to moderately mediate the associations.

## Additional files

Additional file 1: The English language version of questions used to collect the data of importance to be a non-smoker and intention to smoke in childhood. (DOCX 13 kb)

Additional file 2: **Table S1.** Model specification and constraints in life course models. **Table S2.** *P*-values from likelihood ratio tests for the association between SEP determined by area-level disadvantage across the early life span and CDAH-2 smoking status. **Table S3.** Change in effect size after applying combined multiple imputation and inverse probability weighting. (DOCX 27 kb)

## Abbreviations

ASHFS: Australian Schools Health and Fitness Survey
CDAH: Childhood Determinants of Adult Health
CI: Confidence interval
IPW: Inverse probability weight
MI: Multiple imputation
RR: Relative risk
SD: Standard deviation
SEIFA: Socio-economic indexes for areas
SEP: Socioeconomic position
