# Supplementary material for: Socioeconomic position over the life course from childhood and smoking status in mid-adulthood: results from a 25-year follow-up study
Source: BMC Public Health. 2019 Feb 8;19:169. doi: 10.1186/s12889-019-6483-0 (PMC6368790; doi:10.1186/s12889-019-6483-0)
Supplement: Supplementary file 1 — The English language version of questions used to collect the data of importance to be a non-smoker and intention to smoke in childhood. (DOCX 13 kb) [file 12889_2019_6483_MOESM1_ESM.docx]

**Question for assessing the importance to be a non-smoker in childhood**

In your opinion how important is it to you to be a non-smoker?

1. Very important
2. Of some importance
3. Of little importance
4. Not important

**Question for assessing children’s intention to smoke in childhood**

Do you think you will be smoking this time next year?

1. Yes
2. No
3. Don’t know
