# Supplementary material for: Socioeconomic position over the life course from childhood and smoking status in mid-adulthood: results from a 25-year follow-up study
Source: BMC Public Health. 2019 Feb 8;19:169. doi: 10.1186/s12889-019-6483-0 (PMC6368790; doi:10.1186/s12889-019-6483-0)
Supplement: Supplementary file 2 — Table S1. Model specification and constraints in life course models. Table S2. P-values from likelihood ratio tests for the association between SEP determined by area-level disadvantage across the early life span and CDAH-2 smoking status. Table S3. Change in effect size after applying combined multiple imputation and inverse probability weighting. (DOCX 27 kb) [file 12889_2019_6483_MOESM2_ESM.docx]

**Table S1** Model specification and constraints in life course models

|  | Life course model specification | Constraints |
| --- | --- | --- |
| Saturated model | α + β_1_S_1_ + β_2_S_2_ + β_3_S_3_ + θ_12_S_1_S_2_+ θ_13_S_1_S_3_ + θ_23_S_2_S_3_+ θ_123_S_1_S_2_S_3_ |  |
| No effect model | α | β_1_ = β_2_ = β_3_ = θ_12_ = θ_13_ = θ_23_ = θ_123_ = 0 |
| Sensitive period model | α + β_1_S_1_ + β_2_S_2_ + β_3_S_3_ | β_1_ ≠ β_2_ ≠ β_3_, θ_12_ = θ_13_ = θ_23_ = θ_123_ = 0 |
| Critical period model |  |  |
| Baseline, childhood | α + β_1_S_1_ | β_2_ = β_3_ = 0, θ_12_ = θ_13_ = θ_23_ = θ_123_ = 0 |
| CDAH-1, early adulthood | α + β_2_S_2_ | β_1_ = β_3_ = 0, θ_12_ = θ_13_ = θ_23_ = θ_123_ = 0 |
| CDAH-2, middle adulthood | α + β_3_S_3_ | β_1_ = β_2_ = 0, θ_12_ = θ_13_ = θ_23_ = θ_123_ = 0 |
| Accumulation model | α + β_1_S_1_ + β_2_S_2_ + β_3_S_3_ | β_1_ = β_2_ = β_3_ = β, θ_12_ = θ_13_ = θ_23_ = θ_123_ = 0 |
| Social mobility models |  |  |
| Intra-generational (adult) mobility | α + β_2_S_2_ + β_3_S_3_ + θ_23_S_2_S_3_ | θ_23_ = - (β_2_ + β_3_), β_1_ = θ_12_ = θ_13_ = θ_123_ = 0 |
| Any mobility | α + β_1_S_1_ + β_2_S_2_ + β_3_S_3_ + θ_12_S_1_S_2_+ θ_23_ S_2_S_3_ | β_2_ = (β_1_+ β_3_), θ_12_ = θ_23_ = -β_2_, θ_13_ = θ_123_ = 0 |

Si are the binary indicators of socioeconomic position (SEP) at time i, with i=1, 2, 3; Si=0 refers to non-manual SEP at time i while Si=1 refers to manual SEP at time i.

**Table S2** *P*-values from likelihood ratio tests for the association between SEP determined by area-level disadvantage across the early life span and CDAH-2 smoking status, comparing each life course model with the saturated model, Childhood Determinants of Adult Health study, Australia^*^

| Life course model | Smoking status^†^ (N = 2,049) | | Model fit (compared to saturated model) |
| --- | --- | --- | --- |
|  | Former smokers | Current smokers |  |
|  | RR (95% CI) | RR (95% CI) | *P*-value |
| No effect model | - | - | < 0.001 |
| **Sensitive period model** ^‡^ |  |  | **0.493** |
| Relatively disadvantaged, baseline | **0.71 (0.62, 0.83)** | 0.99 (0.81, 1.21) |  |
| Relatively disadvantaged, CDAH-1 | 1.01 (0.86, 1.19) | **1.41 (1.10, 1.80)** |  |
| Relatively disadvantaged, CDAH-2 | 1.09 (0.92, 1.28) | **1.40 (1.10, 1.80)** |  |
| Critical period model |  |  |  |
| Relatively disadvantaged, baseline | 0.73 (0.63, 0.84) | 1.09 (0.89, 1.34) | <0.001 |
| Relatively disadvantaged, CDAH-1 | 1.02 (0.89, 1.17) | 1.68 (1.36, 2.08) | <0.001 |
| Relatively disadvantaged, CDAH-2 | 1.03 (0.90, 1.19) | 1.68 (1.36, 2.07) | <0.001 |
| Accumulation model, No. of times in relatively disadvantaged | 0.95 (0.89, 1.01) | 1.27 (1.15, 1.39) | <0.001 |
| Social mobility model ^§^ |  |  |  |
| Intra-generational (adult) mobility |  |  | <0.001 |
| Stable (a lack of) relative disadvantaged | 1 | 1 |  |
| Moving downwards | 1.05 (0.86, 1.28) | 1.07 (0.79, 1.44) |  |
| Moving upwards | 1.03 (0.83, 1.28) | 1.08 (0.79, 1.46) |  |
| Any mobility |  |  | <0.001 |
| Stable (a lack of) relative disadvantaged/variable | 1 | 1 |  |
| Moving downwards | 1.22 (1.04, 1.43) | 1.21 (0.96, 1.52) |  |
| Moving upwards | 0.79 (0.64, 0.98) | 0.70 (0.51, 0.96) |  |

CDAH: childhood determinants of adult health; RR: relative risk, CI: confidence interval.

^*^ All models were adjusted for age and sex at CDAH-2.

^†^ Relative to never smokers.

^‡^ Life course model in bold is the best-fitting model. RRs (95% CIs) in bold indicate statistically significant results in the best-fitting life course model.

^§^ The intra-generational (adult) mobility model assumes that any downwards change in SEP in adulthood would be harmful to the outcome and any upwards mobility in adulthood would be beneficial, independent of childhood social background. Any mobility model hypothesises that all downward trend changes in the life course are equally harmful to the outcome and all upward shifts are equally beneficial.

**Table S3** Change in effect size after applying combined multiple imputation and inverse probability weighting, Childhood Determinants of Adult Health study, Australia

| Life course model | Former smokers | | Change in RR^*^, % |  | Current smokers | | Change in RR^*^, % |
| --- | --- | --- | --- | --- | --- | --- | --- |
|  | No MI & IPW | MI & IPW |  |  | No MI & IPW | MI & IPW |  |
|  | RR (95% CI) | RR (95% CI) |  |  | RR (95% CI) | RR (95% CI) |  |
| No effect model | - | - |  |  | - | - |  |
| **Sensitive period model** |  |  |  |  |  |  |  |
| Manual, baseline | **0.79 (0.63, 0.98)** | **0.79 (0.63, 0.99)** | 0.3 |  | **1.32 (1.00, 1.73)** | **1.32 (1.00, 1.74)** | -0.4 |
| Manual, CDAH-1 | 1.25 (0.93, 1.68) | 1.25 (0.92, 1.71) | -0.4 |  | 1.42 (0.95, 2.11) | 1.41 (0.88, 2.27) | 0.8 |
| Manual, CDAH-2 | 1.00 (0.73, 1.37) | 1.00 (0.73, 1.38) | <-100^†^ |  | **1.55 (1.04, 2.30)** | 1.55 (0.97, 2.48) | -0.3 |
| Critical period model |  |  |  |  |  |  |  |
| Manual, baseline | 0.82 (0.66, 1.02) | 0.84 (0.67, 1.06) | 14.2 |  | 1.51 (1.15, 1.98) | 1.64 (1.24, 2.18) | -21.3 |
| Manual, CDAH-1 | 1.19 (0.95, 1.48) | 1.25 (0.99, 1.57) | -30.4 |  | 2.00 (1.52, 2.63) | 1.98 (1.48, 2.65) | 1.9 |
| Manual, CDAH-2 | 1.12 (0.88, 1.42) | 1.12 (0.89, 1.42) | -1.2 |  | 2.07 (1.57, 2.74) | 2.08 (1.56, 2.76) | -0.5 |
| **Accumulation model, No. of times manual** | 1.01 (0.91, 1.12) | 1.01 (0.91, 1.12) | -11.0^†^ |  | **1.43 (1.27, 1.61)** | **1.43 (1.27, 1.61)** | 0.1 |
| Social mobility model |  |  |  |  |  |  |  |
| Intra-generational (adult) mobility |  |  |  |  |  |  |  |
| Stable (non-)manual | 1 | 1 |  |  | 1 | 1 |  |
| Moving downwards | 0.96 (0.61, 1.50) | 0.89 (0.54, 1.47) | -<-100^†^ |  | 2.07 (1.33, 3.22) | 2.27 (1.40, 3.68) | -12.8 |
| Moving upwards | 1.14 (0.81, 1.59) | 1.01 (0.70, 1.46) | 92.6^†^ |  | 1.78 (1.19, 2.66) | 2.15 (1.41, 3.29) | -32.9 |
| Any mobility |  |  |  |  |  |  |  |
| Stable (non-)manual/variable | 1 | 1 |  |  | 1 | 1 |  |
| Moving downwards | 1.16 (0.88, 1.54) | 1.08 (0.79, 1.47) | 51.0 |  | 1.52 (1.06, 2.19) | 1.64 (1.10, 2.43) | -17.4 |
| Moving up wards | 0.81 (0.63, 1.04) | 0.81 (0.62, 1.05) | 0.2 |  | 1.09 (0.77, 1.53) | 1.11 (0.77, 1.61) | -29.2 |

MI: multiple imputation, IPW: inverse probability weighting, RR: relative risk, CI: confidence interval, CDAH: childhood determinants of adult health.

^*^ % Change in = (RRb – RRa)/(RRb – 1) * 100. RRb was the average RR before using combined multiple imputation and inverse probability weighting. RRa was the average RR after applying combined multiple imputation and inverse probability weighting.

^‡^ Estimation of % change in RR is unreasonable when the RR was extremely close to 1.
